# Relating quantum coherence and correlations with entropy-based measures

**DOI:** 10.1038/s41598-017-09332-9

**Published:** 2017-09-21

**Authors:** Xiao-Li Wang, Qiu-Ling Yue, Chao-Hua Yu, Fei Gao, Su-Juan Qin

**Affiliations:** 1 State Key Laboratory of Networking and Switching Technology, Beijing University of Posts and Telecommunications, Beijing, 100876 P. R. China; 2 School of Mathematics and Information Science, Henan Polytechnic University, Jiaozuo, 454000 Henan P. R. China; 3 State Key Laboratory of Information Security, Institute of Information Engineering, Chinese Academy of Sciences, Beijing, 100093 P. R. China

## Abstract

Quantum coherence and quantum correlations are important quantum resources for quantum computation and quantum information. In this paper, using entropy-based measures, we investigate the relationships between quantum correlated coherence, which is the coherence between subsystems, and two main kinds of quantum correlations as defined by quantum discord as well as quantum entanglement. In particular, we show that quantum discord and quantum entanglement can be well characterized by quantum correlated coherence. Moreover, we prove that the entanglement measure formulated by quantum correlated coherence is lower and upper bounded by the relative entropy of entanglement and the entanglement of formation, respectively, and equal to the relative entropy of entanglement for all the maximally correlated states.

## Introduction

Quantum coherence arising from quantum superposition^1^, represents a fundamental feature that marks the departure of quantum mechanics from classical physics. Recently, many efforts have been devoted to develop the resource theory of quantum coherence^2–7^. Meanwhile, various properties of quantum coherence have been investigated such as the connections between quantum coherence and quantum correlations in multipartite systems^8–13^, the distillation of coherence^5,14,15^, the dynamics under noisy evolution of quantum coherence^16,17^, among others. The role of coherence in thermodynamics has also been discussed^18,19^.

Besides, quantum coherence in multipartite systems involves the essence of quantum correlations. One of the potential quantum correlations is quantum entanglement^20–24^, which has been widely concerned. Another kind of quantum correlations is quantum discord^25–29^, which may even exist in a separable state with vanished entanglement. Both of them are crucial resources for the development of quantum technologies, such as quantum communication^30,31^, quantum computation^32,33^, quantum metrology^34^, and many more. In these cases, quantum correlations indicate an advantage of quantum methods over classical ones.

Note that previous results in ref.^13^ have established a unified view of quantum discord and quantum entanglement with the framework of quantum coherence based on the *l*_1_–norm of coherence. By contrast, we will adopt the relative entropy of coherence to explore the concise relationships between quantum discord and quantum correlated coherence^13^, which is the coherence between subsystems. This is based on the fact that the entropy plays a crucial role in quantum information theory. Remarkably, quantum correlated coherence can be seen as a ‘correlation function’, which captures the correlation between subsystems. On the other hand, quantum correlated coherence can be used to construct an entanglement measure, which is called the entanglement of coherence (EOC)^13^. However, many important properties, such as additivity, and relations to other entanglement measures, have not been investigated. In this paper, using entropy-based measures, we will show that the EOC is lower and upper bounded by the relative entropy of entanglement and the entanglement of formation, respectively, and equal to the relative entropy of entanglement for maximally correlated states. We also compare the EOC with the entanglement measure, which is the minimal discord over state extensions^35^. Our work provides clear relationships between quantum coherence and correlations with entropy-based measures.

## Results

### Measures of quantum coherence, entanglement and discord

In the framework of coherence theory^4^, let {|*i*〉} be a reference basis in the finite dimensional Hilbert space, and the incoherent sates are those whose density matrices are diagonal in this reference basis, being of the form ${\sum }_{i}\,{p}_{i}|i\rangle \langle i|$, where *p*_*i*_ are probabilities. The set of all incoherent states is denoted as ${\mathcal I}$. It is known that a quantum operation is characterized by a set of Kraus operators {*K*_*l*_} satisfying ${\sum }_{l}\,{K}_{l}^{\dagger }{K}_{l}=I$. In particular, an incoherent quantum operation is that for which there exists a Kraus representation {*K*_*l*_} such that $\frac{{K}_{l}\sigma {K}_{l}^{\dagger }}{Tr({K}_{l}\sigma {K}_{l}^{\dagger })}\in  {\mathcal I}$ for all *l* and all $\sigma \in  {\mathcal I}$. The von Neumann measurement with respect to the reference basis {|*i*〉} (otherwise called the completely dephasing operation^11^) is a special incoherent quantum operation denoted as Π = {|*i*〉 〈*i*|}. For any state *ρ*, we have ${\rm{\Pi }}(\rho )={\rho }^{diag}={\sum }_{i}\,|i\rangle \langle i|\rho |i\rangle \langle i|$. Remarkably, any state *ρ* will generate an incoherent state *ρ* ^*diag*^ by removing all off-diagonal terms from its density matrix in the reference basis under Π. As a quantifier of coherence, we will use the relative entropy of coherence which is defined as ${{\mathscr{C}}}_{re}(\rho )={{\rm{\min }}}_{\sigma \in  {\mathcal I} }\,S(\rho \parallel \sigma )$, where $S(\rho \parallel \sigma )=Tr(\rho \,{\mathrm{log}}_{2}\,\rho )-Tr(\rho \,{\mathrm{log}}_{2}\,\sigma )$ is the quantum relative entropy^36^ and the minimization is taken over the set of incoherent states ${\mathcal I}$. It has been shown that ${{\mathscr{C}}}_{re}(\cdot )$ satisfies all the conditions mentioned in ref.^4^. Crucially, this quantity can be evaluated exactly: ${{\mathscr{C}}}_{re}(\rho )=S({\rho }^{diag})-S(\rho )$, where $S(\rho )=-Tr(\rho \,{\mathrm{log}}_{2}\,\rho )$ is the von Neumann entropy^36^.

In this paper, unless otherwise stated, we will often refer to a bipartite composite quantum system *AB*, where *A* and *B* are local subsystems. For convenience, we say the subsystems *A* and *B* are held by Alice and Bob, respectively. For a given state *ρ*_*AB*_ in system *AB*, the local states of Alice and Bob are denoted as *ρ*_*A*_ = *Tr*_*B*_(*ρ*_*AB*_) and *ρ*_*B*_ = *Tr*_*A*_(*ρ*_*AB*_), respectively, which are obtained by performing a partial trace on *ρ*_*AB*_.

Quantum entanglement^20–24^ is a popular kind of quantum correlations which can not be prepared by local operations and classical communication (LOCC). Any state prepared by LOCC is separable. As a quantifier of entanglement, we will employ the relative entropy of entanglement^21,22^ defined as ${E}_{re}({\rho }_{AB})={{\rm{\min }}}_{\sigma \in {\mathscr{S}}}\,S(\rho \parallel \sigma )$ with the minimization over the set of separable states ${\mathscr{S}}$. Another closely related quantity is entanglement of formation^20^ defined as ${E}_{f}({\rho }_{AB})={{\rm{\min }}}_{\{{p}_{k},|{\psi }_{k}\rangle \}}\,{\sum }_{k}\,{p}_{k}{E}_{re}(|{\psi }_{k}\rangle \langle {\psi }_{k}|)$, where the minimization is taken over all decompositions of the state ${\rho }_{AB}={\sum }_{k}\,{p}_{k}|{\psi }_{k}\rangle \langle {\psi }_{k}|$.

Quantum discord measures the disturbance induced by local measurements to multipartite states^25–29^. Here, we use the discord measure which is based on the entropy theory. Let {|*i*〉_*A*_} and {|*j*〉_*B*_} be some orthonormal bases of subsystems *A* and *B*, respectively. If Bob performs the von Neumann measurement Π_*B*_ = {|*j*〉_*B*_ 〈*j*|} on his subsystem, the post-measurement state of *ρ*_*AB*_ is denoted as

1 $${{\rm{\Pi }}}_{B}({\rho }_{AB})=\sum _{j}\,({I}_{A}\otimes \langle j{|}_{B}\langle j|)\,{\rho }_{AB}({I}_{A}\otimes \langle j{|}_{B}\langle j|),$$

where *I*_*A*_ is the identity operator on subsystem *A*. Then, the asymmetric quantum discord with respect to Π_*B*_ can be written in terms of a difference of relative entropies^25–27^,

2 $${D}_{{{\rm{\Pi }}}_{B}}^{A|B}({\rho }_{AB})=S({\rho }_{AB}\parallel {\rho }_{A}\otimes {\rho }_{B})-S({{\rm{\Pi }}}_{B}({\rho }_{AB})\parallel {\rho }_{A}\otimes {{\rm{\Pi }}}_{B}({\rho }_{B})).$$

In the classical-quantum dichotomy, to remove the measurement-basis dependence, the asymmetric quantum discord is defined as ${D}^{A|B}({\rho }_{AB})\equiv {{\rm{\min }}}_{{{\rm{\Pi }}}_{B}}\,{D}_{{{\rm{\Pi }}}_{B}}^{A|B}({\rho }_{AB})$ with the minimization over all local von Neumann measurements Π_*B*_. If Alice only performs the von Neumann measurement Π_*A*_ = {|*i*〉_*A*_ 〈*i*|} on her subsystem, the similar results are available. Besides, if both Alice and Bob perform local von neumannn measurements Π_*A*_ and Π_*B*_ on their respective subsystems, the symmetric quantum discord (global quantum discord in bipartite system^27^) with respect to ${{\rm{\Pi }}}_{A}\otimes {{\rm{\Pi }}}_{B}$ is defined as:

$${D}_{{{\rm{\Pi }}}_{A}\otimes {{\rm{\Pi }}}_{B}}({\rho }_{AB})=S({\rho }_{AB}\parallel {\rho }_{A}\otimes {\rho }_{B})-S({{\rm{\Pi }}}_{A}\otimes {{\rm{\Pi }}}_{B}({\rho }_{AB})\parallel {{\rm{\Pi }}}_{A}({\rho }_{A})\otimes {{\rm{\Pi }}}_{B}({\rho }_{B})).$$

Then, the symmetric quantum discord is defined as $D({\rho }_{AB})\equiv {{\rm{\min }}}_{{{\rm{\Pi }}}_{A}\otimes {{\rm{\Pi }}}_{B}}\,{D}_{{{\rm{\Pi }}}_{A}\otimes {{\rm{\Pi }}}_{B}}\,({\rho }_{AB})$, with the minimization over all the local von Neumann measurements of Alice and Bob.

Recently, Yadin *et al*.^37^ studied the asymmetric basis-dependent discord ${D}_{{{\rm{\Pi }}}_{B}}^{A|B}(\cdot )$, which can be seen as the basis-dependent measure of quantumness of correlation, and the properties of ${D}_{{{\rm{\Pi }}}_{B}}^{A|B}(\cdot )$ under the strictly incoherent operations were investigated. Here, we will connect the basis-dependent discord and quantum correlated coherence.

### Quantum correlated coherence and quantum discord

Let {|*i*〉_*A*_} and {|*j*〉_*B*_} be the local reference bases of subsystems *A* and *B*, respectively, and we usually use their tensor product {|*ij*〉_*AB*_} as the reference basis of the composite system *AB*. For a state *ρ*_*AB*_ in system *AB*, its total coherence is ${{\mathscr{C}}}_{re}({\rho }_{AB})$, while ${{\mathscr{C}}}_{re}({\rho }_{A})$ and ${{\mathscr{C}}}_{re}({\rho }_{B})$ are known as local coherences. Whenever *ρ*_*AB*_ is a product state, the sum of local coherences is equal to the total coherence. Generally, the relative entropy of coherence admits the super-additive property^11^:

3 $${{\mathscr{C}}}_{re}({\rho }_{AB})\ge {{\mathscr{C}}}_{re}({\rho }_{A})+{{\mathscr{C}}}_{re}({\rho }_{B}).$$

Thus, the definition of quantum correlated coherence with respect to the relative entropy of coherence is given in the following.

**Definition 1**. (*K*. *C*. *Tan et al*.^13^) *Let* {|*i*〉_*A*_} *and* {|*j*〉_*B*_} *be the local reference bases of subsystems A and B*, *respectively*. *For a given state ρ*_*AB*_ *in system AB*, *its quantum correlated coherence is defined as*

4 $${{\mathscr{C}}}_{re}^{cc}({\rho }_{AB})\equiv {{\mathscr{C}}}_{re}({\rho }_{AB})-{{\mathscr{C}}}_{re}({\rho }_{A})-{{\mathscr{C}}}_{re}({\rho }_{B}).$$

Obviously, quantum correlated coherence is the total coherence between subsystems and non-negative. In fact, quantum correlated coherence is a ‘correlation function’, which is similar as quantum mutual information^36^. For a state *ρ*_*AB*_, whatever the reference bases of subsystems are, its quantum correlated coherence is always zero, if and only if *ρ*_*AB*_ has no correlations i.e., ${\rho }_{AB}={\rho }_{A}\otimes {\rho }_{B}$. The ‘only if’ part is directly derived from the Theorem 1 below. In this sense, quantum correlated coherence can be seen as the basis-dependent measure of quantumness of correlation and accounts for quantum correlations, for example, quantum discord.

With respect to the local reference bases of {|*i*〉_*A*_} and {|*j*〉_*B*_} of subsystems *A* and *B*, respectively, the local von Neumann measurements of Alice and Bob are denoted as Π_*A*_ = {|*i*〉_*A*_ 〈*i*|} and Π_*B*_ = {|*j*〉_*B*_ 〈*j*|}, respectively. Π_*A*_ and Π_*B*_ are also called completely resource (coherence) destroying maps which play a crucial role in the resource theory of coherence^38^. By direct calculation, we get that the consumption of quantum correlated coherence for any state *ρ*_*AB*_ under Π_*B*_ coincides with the asymmetric basis-dependent discord ${D}_{{{\rm{\Pi }}}_{B}}^{A|B}({\rho }_{AB})$, i.e., ${{\mathscr{C}}}_{re}^{cc}({\rho }_{AB})-{{\mathscr{C}}}_{re}^{cc}({{\rm{\Pi }}}_{B}({\rho }_{AB}))={D}_{{{\rm{\Pi }}}_{B}}^{A|B}({\rho }_{AB})$. According to the condition for the asymmetric basis-dependent discord ${D}_{{{\rm{\Pi }}}_{B}}^{A|B}({\rho }_{AB})$ to vanish^37^, we have the following result.

**Proposition 1**. Let {|i〉_A_} and {|j〉_B_} be the local reference bases of subsystems A and B, respectively, and the local von Neumann measurement in the basis {|j〉_B_} is denoted as Π_B_. For a given state ρ_AB_ in system AB, its quantum correlated coherence remains unchanged under Π_B_, i.e., ${{\mathscr{C}}}_{re}^{cc}({\rho }_{AB})={{\mathscr{C}}}_{re}^{cc}({{\rm{\Pi }}}_{B}({\rho }_{AB}))$, if and only if there exists a decomposition ${\rho }_{AB}={\sum }_{\alpha }\,{p}_{\alpha }{\rho }_{A}^{\alpha }\otimes {\rho }_{B}^{\alpha }$ such that p_α_ are probabilities and all the states ${\rho }_{B}^{\alpha }$ are perfectly distinguishable by the von Neumann measurement in the reference basis {|j〉_B_}.

In Proposition 1, two different states, which are perfectly distinguishable by the von Neumann measurement in the reference basis {|*j*〉_*B*_}, must have disjoint coherence support. The coherence support of a state is the set of some incoherent basis vectors which have nonzero overlap with the state^37^. Proposition 1 provides a concise relationship between quantum correlated coherence and quantum-classical states^29^ for some local reference bases of subsystems *A* and *B*.

Using the very similar arguments as ${D}_{{{\rm{\Pi }}}_{B}}^{A|B}({\rho }_{AB})$, we obtain that quantum correlated coherence is corresponding to the symmetric basis-dependent discord ${{\mathscr{C}}}_{re}^{cc}({\rho }_{AB})={D}_{{{\rm{\Pi }}}_{A}\otimes {{\rm{\Pi }}}_{B}}({\rho }_{AB})$. Moreover, we also find the condition for quantum correlated coherence (the symmetric basis-dependent discord) to vanish.

**Theorem 1**. Let {|i〉_A_} and {|j〉_B_} be the local reference bases of subsystems A and B, respectively. For a given state ρ_AB_ in system AB, its quantum correlated coherence is equal to zero, i.e., ${{\mathscr{C}}}_{re}^{cc}({\rho }_{AB})=0$, if and only if there exists a decomposition,

5 $${\rho }_{AB}=\sum _{k,l}\,{p}_{kl}{\rho }_{A}^{k}\otimes {\rho }_{B}^{l},$$

*such that p*_*kl*_ *are probabilities*, *and all the states* ${\rho }_{A}^{k}$ *and* ${\rho }_{B}^{l}$ *are perfectly distinguishable by the local von Neumann measurements with respect to the local reference bases* {|*i*〉_*A*_} *and* {|*j*〉_*B*_}, *respectively*.

Proof. To prove the sufficiency, we will use the following property of von Neumann entropy^36^,

6 $$S(\sum _{i}\,{p}_{i}{\rho }_{i})=h(\{{p}_{i}\})+\sum _{i}\,{p}_{i}S({\rho }_{i}),$$

where *h*({*p*_*i*_}) is Shannon entropy and all *ρ*_*i*_ have support on orthogonal subspaces. Since all ${\rho }_{A}^{k}$ and ${\rho }_{B}^{l}$ are perfectly distinguishable by the local von Neumann measurements in the local reference bases {|*i*〉_*A*_} and {|*j*〉_*B*_}, respectively, $\{{\rho }_{A}^{k(l)}\}$, $\{{\rho }_{A}^{k}\otimes {\rho }_{B}^{l}\}$, $\{{\rho }_{A}^{k(l)diag}\}$, and $\{{\rho }_{A}^{kdiag}\otimes {\rho }_{B}^{ldiag}\}$ are sets of states with support on orthogonal subspaces. Direct calculation shows that ${{\mathscr{C}}}_{re}^{cc}({\rho }_{AB})=0$.

Note that ${{\mathscr{C}}}_{re}^{cc}({\rho }_{AB})=S({\rho }_{AB}\parallel {\rho }_{A}\otimes {\rho }_{B})$ − $S({{\rm{\Pi }}}_{A}\otimes {{\rm{\Pi }}}_{B}({\rho }_{AB})\parallel {{\rm{\Pi }}}_{A}({\rho }_{A})\otimes {{\rm{\Pi }}}_{B}({\rho }_{B}))$, where Π_*A*_ and Π_*B*_ are the local von Neumann measurements in the local reference bases {|*i*〉_*A*_} and {|*j*〉_*B*_}, respectively. To prove the necessity, we will use the condition for the quantum relative entropy which is unchanged under a quantum operation^39,40^ and the explicit proof is left to the Methods.

Theorem 1 has several implications. First, it implies that a state with vanished quantum correlated coherence is a specially classical-classical state^41^ but not necessary to be a bipartite incoherent state^8,13^. Particularly, a qubit-qubit state with vanished quantum correlated coherence is a product states or a bipartite incoherent state. More complex cases only emerge in higher dimension. For example, the following qutrit-qutrit state with vanished quantum correlated coherence, has yet local coherences:

$${\rho }_{AB}=\tfrac{1}{2}(\tfrac{1}{2}|0\rangle \langle 0|+\tfrac{1}{2}|{+}_{01}\rangle \langle {+}_{01}|)\otimes (\tfrac{2}{3}|0\rangle \langle 0|+\tfrac{1}{3}|{+}_{02}\rangle \langle {+}_{02}|)+\tfrac{1}{2}|2\rangle \langle 2|\otimes |1\rangle \langle 1|,$$

where $|{+}_{ij}\rangle =\tfrac{1}{\sqrt{2}}(|i\rangle +|j\rangle )$ and the reference basis of each subsystem is the computable basis. Second, Theorem 1 indicates the structure of bipartite states which satisfy the super-additive property of the relative entropy of coherence with equality. This settles an important question left open in previous literature^11,42^ that whether the equality of Eq. (3) holds if and only if *ρ*_*AB*_ is product or incoherent. Finally, if we choose the local eigenbases of *ρ*_*A*_ and *ρ*_*B*_ as the reference bases of subsystems *A* and *B*, respectively, Proposition 1 and Theorem 1 reduce to the corresponding results in ref.^13^. In this sense, our results somewhat generalize the previous results.

The above results show that quantum correlated coherence and the basis-dependent discord are closely related. Due to the equality ${{\mathscr{C}}}_{re}^{cc}({\rho }_{AB})={D}_{{{\rm{\Pi }}}_{A}\otimes {{\rm{\Pi }}}_{B}}({\rho }_{AB})$, the symmetric quantum discord is rewritten as $D({\rho }_{AB})={{\rm{\min }}}_{\{{|i\rangle }_{A},{|j\rangle }_{B}\}}\,{{\mathscr{C}}}_{re}^{cc}({\rho }_{AB})$. Recall that the symmetric quantum discord based on the pseudo distance of relative entropy is equal to the basis-free quantum coherence^12,28^, which is denoted as ${{\mathscr{C}}}^{free}({\rho }_{AB})={{\rm{\min }}}_{\{{|i\rangle }_{A},{|j\rangle }_{B}\}}\,{{\mathscr{C}}}_{re}({\rho }_{AB})$. Both of the minimization are taken over all the local generic bases {|*i*〉_*A*_} and {|*j*〉_*B*_} of subsystems *A* and *B*, respectively. However, one may also consider defining a discord measure *D*_*POVM*_(·) via general local positive-operator-valued measurements (POVMs) on each subsystem^25,29^ instead of the local von Neumann measurements. Comparing these three quantifiers of quantum discord, we easily have the inequality ${{\mathscr{C}}}^{free}({\rho }_{AB})\ge D({\rho }_{AB})\ge {D}_{POVM}({\rho }_{AB})$. Whenever these three quantities are zero, the corresponding states are classical-classical states^26,41^. Similarly, the asymmetric quantum discord *D* ^*A*|*B*^(*ρ*_*AB*_) can also be represented by quantum correlated coherence.

In multipartite systems, the global quantum discord (GQD)^27^ can even be represented with quantum correlated coherence. It is worth noting that the reference basis of a multipartite system is the tensor product of the local reference bases of all the subsystems. For a *N*-partite state ${\rho }_{{C}_{1}{C}_{2}\cdots {C}_{N}}$, its GQD is represented as $D({\rho }_{{C}_{1}{C}_{2}\cdots {C}_{N}})={{\rm{\min }}}_{\{{|{i}_{1}\rangle }_{{C}_{1}},{|{i}_{2}\rangle }_{{C}_{2}},\cdots ,{|{i}_{N}\rangle }_{{C}_{N}}\}}\,{{\mathscr{C}}}_{re}^{cc}({\rho }_{{C}_{1}{C}_{2}\cdots {C}_{N}})$, where the minimization is taken over all the generic bases of the multipartite system. With respect to some reference basis of the *N*-partite system, $\{{|{i}_{1}\rangle }_{{C}_{1}}{|{i}_{2}\rangle }_{{C}_{2}}\cdots {|{i}_{N}\rangle }_{{C}_{N}}\}$, it holds that ${{\mathscr{C}}}_{re}^{cc}({\rho }_{{C}_{1}{C}_{2}\cdots {C}_{N}})={{\mathscr{C}}}_{re}({\rho }_{{C}_{1}{C}_{2}\cdots {C}_{N}})-{\sum }_{i}\,{{\mathscr{C}}}_{re}({\rho }_{{C}_{i}})$. Using the super-additive property of the relative entropy of coherence given in inequality (3), we are easy to get that the GQD of any *N*-partite state is non-negative and for a multipartite classical state it is equal to zero. This provides a simple proof of the non-negativity of the GQD in ref.^27^. These results mean that quantum discord in multipartite systems can be better understood with the framework of quantum coherence.

### Quantum correlated coherence and quantum entanglement

According to the above discussion, we know that if for arbitrary local reference bases of subsystems *A* and *B*, the quantum correlated coherence of *ρ*_*AB*_ does not vanish, there must exist quantum correlation (quantum discord) between subsystems *A* and *B*. Moreover, it is also possible to characterize entanglement with quantum correlated coherence via state extensions, for example, the entanglement of coherence (EOC)^13^. Then, entanglement can be seen as the irreducible residue of quantum correlated coherence. This highlights the non-locality of quantum entanglement.

In the following, we will discuss some properties of the EOC. For a given state *ρ*_*AB*_ in system *AB*, a bipartite state *ρ*_*AA*′*BB*′_ is an extension of *ρ*_*AB*_ if *ρ*_*AA*′*BB*′_ satisfies *Tr*_*A*′*B*′_(*ρ*_*AA*′*BB*′_) = *ρ*_*AB*_, where subsystems *AA*′ and *BB*′ are held by Alice and Bob, respectively^13,43^. The following definition of the EOC establish a connection between entanglement and quantum correlated coherence.

**Definition 2**. (*K*. *C*. *Tan et al*.^13^) *For a given state ρ*_*AB*_, *ρ*_*AA*′*BB*′_ *is its unitarily symmetric extension and let the local eigenbases of ρ*_*AA*′_ *and ρ*_*BB*′_ *be the local reference bases of subsystems AA*′ *and BB*′, *respectively*. *The entanglement of coherence* (*EOC*) *of ρ*_*AB*_ *is defined as*

7 $${E}_{re}^{cc}({\rho }_{AB})\equiv \,{\rm{\min }}\,{{\mathscr{C}}}_{re}^{cc}({\rho }_{AA^{\prime} BB^{\prime} }),$$

*where the minimization is taken over all possible unitarily symmetric extensions ρ*_*AA*′*BB*′_.

In Definition 2, the extension *ρ*_*AA*′*BB*′_ is unitarily symmetric if it remains invariant up to local unitary operations on *AA*′ and *BB*′ under a system swap between Alice and Bob. It has been shown that the EOC has the properties^13^: non-negative, vanished for separated states, invariant under local unitary operations, non-increasing under LOCC operations, and convex. Furthermore, using entropy-based measures, we give the bounds of the EOC.

**Theorem 2**. For a given state ρ_AB_, it holds that

8 $${E}_{re}({\rho }_{AB})\le {E}_{re}^{cc}({\rho }_{AB})\le {E}_{f}({\rho }_{AB}\mathrm{).}$$

*If ρ*_*AB*_ *is a pure state*, *these three quantities in inequality* (8) *are equal*.

Proof. Taking some unitarily symmetric extension *ρ*_*AA*′*BB*′_ of *ρ*_*AB*_, we have

9 $${{\mathscr{C}}}_{re}^{cc}({\rho }_{AA^{\prime} BB^{\prime} })={{\mathscr{C}}}_{re}({\rho }_{AA^{\prime} BB^{\prime} })\ge {E}_{re}({\rho }_{AA^{\prime} BB^{\prime} })\ge {E}_{re}({\rho }_{AB}),$$

where the first inequality is due to that the relative entropy of coherence is no less than the relative entropy of entanglement for a state^12^, and the last inequality is due to that entanglement is un-increased under LOCC operations^21,22^. Then, the inequality (9) means that ${E}_{re}({\rho }_{AB})\le {E}_{re}^{cc}({\rho }_{AB})$.

To prove the inequality ${E}_{re}^{cc}({\rho }_{AB})\le {E}_{f}({\rho }_{AB})$, we consider the optimal decomposition of the state ${\rho }_{AB}={\sum }_{i}\,{p}_{i}^{\ast }|{\psi }_{i}^{\ast }\rangle \langle {\psi }_{i}^{\ast }|$ such that ${E}_{f}({\rho }_{AB})={\sum }_{i}\,{p}_{i}^{\ast }{E}_{re}(|{\psi }_{i}^{\ast }\rangle \langle {\psi }_{i}^{\ast }|)$. Every state $|{\psi }_{i}^{\ast }\rangle$ is represented with the Schmidt decomposition $|{\psi }_{i}^{\ast }\rangle ={\sum }_{{j}_{i}}\,{\lambda }_{{j}_{i}}{|{j}_{i}\rangle }_{A}{|{j}_{i}\rangle }_{B}$. For every *i*, {|*j*_*i*_〉_*A*_} and {|*j*_*i*_〉_*B*_} are expanded to be the orthonormal bases of subsystems *A* and *B*, respectively, but both of them are still labeled with original symbols. Define the state

$${\rho }_{AA^{\prime} BB^{\prime} }^{{\rm{\Delta }}}\equiv \sum _{i}\,{p}_{i}^{\ast }\sum _{{j}_{i},{j}_{i}^{^{\prime} }}\,{\lambda }_{{j}_{i}}{\lambda }_{{j}_{i}^{^{\prime} }}{|{j}_{i}\rangle }_{A}{\langle {j}_{i}^{^{\prime} }|\otimes |i\rangle }_{A^{\prime} }{\langle i|\otimes |{j}_{i}\rangle }_{B}{\langle {j}_{i}^{^{\prime} }|\otimes |i\rangle }_{B^{\prime} }\langle i|,$$

where {|*i*〉_*A*′(*B*′)_} is the orthonormal basis of system *A*′(*B*′). Note that {|*j*_*i*_〉_*A*_|*i*〉_*A*′_} and {|*j*_*i*_〉_*B*_|*i*〉_*B*′_} are local eigenbases of *ρ*_*AA*′_ and *ρ*_*BB*′_, respectively. Let ${U}_{AA^{\prime} }={\sum }_{i,{j}_{i}}\,{|{j}_{i}\rangle }_{BA}{\langle {j}_{i}|\otimes |i\rangle }_{B^{\prime} A^{\prime} }\langle i|$ and ${U}_{BB^{\prime} }={\sum }_{i,{j}_{i}}\,{|{j}_{i}\rangle }_{AB}{\langle {j}_{i}|\otimes |i\rangle }_{A^{\prime} B^{\prime} }\langle i|$ and a little thought shows that these two unitary operators satisfy

$${U}_{AA^{\prime} }\otimes {U}_{BB^{\prime} }({T}_{swap}{\rho }_{AA^{\prime} BB^{\prime} }^{{\rm{\Delta }}}{T}_{swap}^{\dagger })\,{U}_{AA^{\prime} }^{\dagger }\otimes {U}_{BB^{\prime} }^{\dagger }={\rho }_{AA^{\prime} BB^{\prime} }^{{\rm{\Delta }}},$$

where *T*_*swap*_ denotes the swap operator with respect to the local eigenbases of *ρ*_*AA*′_ and *ρ*_*BB*′_, i.e., {|*j*_*i*_〉_*A*_|*i*〉_*A*′_} and {|*j*_*i*_〉_*B*_|*i*〉_*B*′_}. Therefore, *ρ*_*AA*′*BB*′_ is unitarily symmetric. Consequently, we calculate the quantum correlated coherence of ${\rho }_{AA^{\prime} BB^{\prime} }^{{\rm{\Delta }}}$,

$$\begin{array}{rcl}{{\mathscr{C}}}_{re}^{cc}({\rho }_{AA^{\prime} BB^{\prime} }^{{\rm{\Delta }}}) & = & {{\mathscr{C}}}_{re}({\rho }_{AA^{\prime} BB^{\prime} }^{{\rm{\Delta }}})\\  & = & \sum _{i}\,{p}_{i}^{\ast }S(\sum _{{j}_{i}}\,{\lambda }_{{j}_{i}}^{2}{|{j}_{i}\rangle }_{A}{\langle {j}_{i}|\otimes |{j}_{i}\rangle }_{B}\langle {j}_{i}|)\\  & = & \sum _{i}\,{p}_{i}^{\ast }S(\sum _{{j}_{i}}\,{\lambda }_{{j}_{i}}^{2}{|{j}_{i}\rangle }_{A}\langle {j}_{i}|)\\  & = & {E}_{f}({\rho }_{AB}),\end{array}$$

where the second equality is due to the property of von Neumann entropy given in Eq. (6). The above equality implies that ${E}_{re}^{cc}({\rho }_{AB})\le {{\mathscr{C}}}_{re}^{cc}({\rho }_{AA^{\prime} BB^{\prime} }^{{\rm{\Delta }}})={E}_{f}({\rho }_{AB})$. If *ρ*_*AB*_ is a pure state, its relative entropy of entanglement is equal to its entanglement of formation, and then equal to its EOC. Hence, the desired results of Theorem 2 are obtained.□

From Theorem 2, we conclude that the EOC is not strictly less than the relative entropy of entanglement for a bipartite state, since for pure states they are equal. Moreover, for a maximally correlated state^44^, it is of the form:

10 $${\rho }_{AB}=\sum _{s,t}\,{\rho }_{st}{|s\rangle }_{A}{\langle t|\otimes |s\rangle }_{B}\langle t|,$$

where {|*s*〉_*A*_} and {|*t*〉_*B*_} are some orthonormal bases of subsystems *A* and *B* respectively and *ρ*_*st*_ are the matrix elements. Then, its EOC is also equal to its relative entropy of entanglement. We show this result in the following theorem.

**Theorem 3**. *For any maximally correlated state ρ*_*AB*_ *as given in* Eq. (10), *its EOC is equal to its relative entropy of entanglement*, *i*.*e*., ${E}_{re}^{cc}({\rho }_{AB})={E}_{re}({\rho }_{AB})$.

Proof: For the maximally correlated state *ρ*_*AB*_, it has the form given in Eq. (10). Let the local eigenbases of *ρ*_*A*_ and *ρ*_*B*_, i.e., {|*s*〉_*A*_} and {|*t*〉_*B*_}, be the local reference bases of subsystems *A* and *B*, respectively. According to the ref.^8^,we have ${{\mathscr{C}}}_{re}({\rho }_{A}^{\ast })={E}_{re}({\rho }_{AB})$, where ${\rho }_{A}^{\ast }={\sum }_{s,t}\,{\rho }_{st}{|s\rangle }_{A}\langle t|$ in subsystem *A*. Direct calculation yields ${{\mathscr{C}}}_{re}^{cc}({\rho }_{AB})=S({\rho }_{AB}^{diag})-S({\rho }_{AB})$ = $S({\rho }_{A}^{\ast diag})-S({\rho }_{A}^{\ast })={{\mathscr{C}}}_{re}({\rho }_{A}^{\ast })$. Obviously, ${\rho }_{AB}\otimes {|00\rangle }_{A^{\prime} B^{\prime} }\langle 00|$ ia a unitarily symmetric extension of *ρ*_*AB*_. With respect to the local eigenbases of *ρ*_*AA*′_ and *ρ*_*BB*′_ as the fixed bases of subsystems *AA*′ and *BB*′, respectively, we have the equality ${{\mathscr{C}}}_{re}^{cc}({\rho }_{AB}\otimes {|00\rangle }_{A^{\prime} B^{\prime} }\langle 00|)={{\mathscr{C}}}_{re}^{cc}({\rho }_{AB})$. Then, it holds that ${E}_{re}^{cc}({\rho }_{AB})\le {{\mathscr{C}}}_{re}^{cc}({\rho }_{AB}\otimes {|00\rangle }_{A^{\prime} B^{\prime} }\langle 00|)={{\mathscr{C}}}_{re}^{cc}({\rho }_{AB})$. Combining the aforementioned results and Theorem 2, we arrive at the result ${E}_{re}^{cc}({\rho }_{AB})={E}_{re}({\rho }_{AB})$.□

Using the proof of Theorem 3, we confirm that for a maximally correlated state *ρ*_*AB*_, its EOC is even equal to its quantum correlated coherence with respect to the local eigenbases of *ρ*_*A*_ and *ρ*_*B*_, respectively. Moreover, with Theorem 3, it is easy to find a state for which the EOC is strictly less than the entanglement of formation, for example, the maximally correlated Bell diagonal state in the two-qubit system^20,21^, ${\rho }_{AB}^{mc}=\frac{3}{4}|{{\rm{\Phi }}}^{+}\rangle \langle {{\rm{\Phi }}}^{+}|+\frac{1}{4}|{{\rm{\Phi }}}^{-}\rangle \langle {{\rm{\Phi }}}^{-}|$, where $|{{\rm{\Phi }}}^{\pm }\rangle =\frac{1}{\sqrt{2}}(|00\rangle \pm |11\rangle )$. However, we do not know whether the EOC is equal to the relative entropy of entanglement for any mixed state. In addition, for any bipartite state *ρ*_*AB*_ and *τ*_*CD*_, the EOC satisfies the following sub-additivity,

$${\rm{\max }}\,\{{E}_{re}^{cc}({\rho }_{AB}),{E}_{re}^{cc}({\tau }_{CD})\}\le {E}_{re}^{cc}({\rho }_{AB}\otimes {\tau }_{CD})\le {E}_{re}^{cc}({\rho }_{AB})+{E}_{re}^{cc}({\tau }_{CD}\mathrm{).}$$

An alternative measure of entanglement formulated by quantum correlated coherence (quantum discord) is defined as ${\bar{E}}_{re}^{cc}({\rho }_{AB})\equiv \,{\rm{\min }}\,D({\rho }_{AA^{\prime} BB^{\prime} })$, where the minimization is taken over all possible unitarily symmetric extensions *ρ*_*AA*′*BB*′_ of *ρ*_*AB*_, and $D({\rho }_{AA^{\prime} BB^{\prime} })={{\rm{\min }}}_{\{{|i\rangle }_{AA^{\prime} },{|j\rangle }_{BB^{\prime} }\}}\,{{\mathscr{C}}}_{re}^{cc}({\rho }_{AA^{\prime} BB^{\prime} })$, with the minimization over all reference bases {|*i*〉_*AA*′_} and {|*j*〉_*BB*′_} of subsystems *AA*′ and *BB*′, respectively. Removing the the property of unitary symmetry of extension *ρ*_*AA*′*BB*′_ in the definition ${\bar{E}}_{re}^{cc}({\rho }_{AB})$, we denote this new measure of entanglement as ${\tilde{E}}_{re}^{cc}({\rho }_{AB})$. Remarkably, ${\tilde{E}}_{re}^{cc}({\rho }_{AB})$ is equivalent to the entanglement measure *E* which is the minimal discord over state extensions^35^. Moreover, ${\bar{E}}_{re}^{cc}({\rho }_{AB})$ and ${\tilde{E}}_{re}^{cc}({\rho }_{AB})$ have the properties: non-negative, vanished for separated states, invariant under local unitary operations, non-increasing under local operations, convex and upper bounded by entanglement of formation *E*_*f*_ (*ρ*_*AB*_). However, the properties of ${\bar{E}}_{re}^{cc}({\rho }_{AB})$ and ${\tilde{E}}_{re}^{cc}({\rho }_{AB})$, which are the invariance (non-increasing property) under classical communication and the relation to the relative entropy of entanglement, are not clear. In this sense, the EOC is more advantageous than ${\bar{E}}_{re}^{cc}({\rho }_{AB})$ and ${\tilde{E}}_{re}^{cc}({\rho }_{AB})$.

In multipartite systems, there exists an entanglement measure like the definition of EOC. For a *N*-partite state ${\rho }_{{C}_{1}{C}_{2}\cdots {C}_{N}}$, its entanglement of coherence is defined as ${E}_{re}^{cc}({\rho }_{{C}_{1}{C}_{2}\cdots {C}_{N}})\equiv \,{\rm{\min }}\,{{\mathscr{C}}}_{re}^{cc}({\rho }_{{C}_{1}{C}_{1}^{^{\prime} }{C}_{2}{C}_{2}^{^{\prime} }\cdots {C}_{N}{C}_{N}^{^{\prime} }})$, where the minimization is taken over all possible unitarily symmetric extensions ${\rho }_{{C}_{1}{C}_{1}^{^{\prime} }{C}_{2}{C}_{2}^{^{\prime} }\cdots {C}_{N}{C}_{N}^{^{\prime} }}$ of ${\rho }_{{C}_{1}{C}_{2}\cdots {C}_{N}}$, $T{r}_{{C}_{1}^{^{\prime} }{C}_{2}^{^{\prime} }\cdots {C}_{N}^{^{\prime} }}({\rho }_{{C}_{1}{C}_{1}^{^{\prime} }{C}_{2}{C}_{2}^{^{\prime} }\cdots {C}_{N}{C}_{N}^{^{\prime} }})={\rho }_{{C}_{1}{C}_{2}\cdots {C}_{N}}$, and the local fixed bases are the eigenbases of ${\rho }_{{C}_{1}{C}_{1}^{^{\prime} }}$, ${\rho }_{{C}_{2}{C}_{2}^{^{\prime} }}$, …, and ${\rho }_{{C}_{N}{C}_{N}^{^{\prime} }}$, respectively. Note that the extension ${\rho }_{{C}_{1}{C}_{1}^{^{\prime} }{C}_{2}{C}_{2}^{^{\prime} }\cdots {C}_{N}{C}_{N}^{^{\prime} }}$ is unitarily symmetric if it remains invariant up to local unitary operations on ${C}_{i}{C}_{i}^{^{\prime} }$ and ${C}_{j}{C}_{j}^{^{\prime} }$ under a system swap between ${C}_{i}{C}_{i}^{^{\prime} }$ and ${C}_{j}{C}_{j}^{^{\prime} }$ for any *i*, *j* = 1, 2, …, *N*. Referring to the proofs of EOC as an entanglement measure^13^, we can show that ${E}_{re}^{cc}({\rho }_{{C}_{1}{C}_{2}\cdots {C}_{N}})$ has the properties: non-negative and vanished for separated states, invariant under local unitary operations, non-increasing under LOCC operations, and convex. These results show that the entanglement in multipartite systems can also be characterized by quantum correlated coherence via state extensions.

## Discussion

In this paper, using entropy-based measures, we have obtained the concise relationships between quantum coherence and quantum discord as well as quantum entanglement. The results mean that quantum discord and entanglement can be well characterized by quantum correlated coherence. In particular, we gave the condition for quantum correlated coherence (symmetric basis-dependent discord)to vanish, and this condition provides the explicit structure of states which satisfy the super-additive property of the relative entropy of coherence with equality. We further proved the lower and upper bounds of the EOC and showed that the EOC is equal to the relative entropy of entanglement in a large number of scenarios including all pure states and maximally correlated states. For pure states, the LOCC monotonicity (monotonicity on average under LOCC operations^24,45^) of EOC is easily obtained with Theorem 2. However, we do not know whether the EOC of a general mixed state is LOCC monotone^24,45^, and we leave it open for future research. Finally, we also generalized our results to multipartite settings.

Quite remarkably, one-way basis-dependent quantum deficit in the bibapartite quantum system is equal to the amount of the total coherence lost by the von Neumann measurement with respect to the reference basis of one of the subsystems^11^. These results suggest that the quantum properties of correlations originate from the quantum properties of coherence and quantum correlations can be unified understood within the framework of coherence. We hope that this work is helpful for further understanding quantum correlations and developing quantum technologies.

## Methods

### Proof of Theorem 1 in the main text

Here, we prove that a state *ρ*_*AB*_ with vanished quantum correlated coherence has a decomposition given in Eq. (5) in the main text.

For a given state *ρ*_*AB*_ with vanished quantum correlated coherence, its symmetric quantum discord is equal to zero, i.e., *D*(*ρ*_*AB*_) = 0. Then, *ρ*_*AB*_ is a classical-classical state^40^ with the form

11 $${\rho }_{AB}=\sum _{\alpha ,\beta }\,{\lambda }_{\alpha \beta }|{\psi }_{\alpha }\rangle \langle {\psi }_{\alpha }|\otimes |{\varphi }_{\beta }\rangle \langle {\varphi }_{\beta }|,$$

where {|*ψ*_*α*_〉} and {|*ϕ*_*β*_〉} are orthonormal bases of subsystems *A* and *B*, respectively. From the main text, we see that

12 $${{\mathscr{C}}}_{re}^{cc}({\rho }_{AB})=0\iff S({\rho }_{AB}\parallel {\rho }_{A}\otimes {\rho }_{B})=S({{\rm{\Pi }}}_{A}\otimes {{\rm{\Pi }}}_{B}({\rho }_{AB})\parallel {{\rm{\Pi }}}_{A}({\rho }_{A})\otimes {{\rm{\Pi }}}_{B}({\rho }_{B})).$$

where the von Neumann measurements Π_*A*_ and Π_*B*_ are with respect to the local reference bases {|*i*〉_*A*_} and {|*j*〉_*B*_} of subsystems *A* and *B*, respectively.

Recall that the quantum relative entropy is unchanged under a quantum operation ${\mathcal E}$, i.e., $S(\rho \parallel \sigma )=S( {\mathcal E} (\rho )\parallel  {\mathcal E} (\sigma ))$, if and only if there is a recovery operation ${\mathcal R}$ satisfying^39^: ${\mathcal R} \circ  {\mathcal E} (\rho )=\rho$, ${\mathcal R} \circ  {\mathcal E} (\sigma )=\sigma$. Moreover, there is an explicit formula for the recovery operation: ${\mathcal R} (X)={\sigma }^{\tfrac{1}{2}}{ {\mathcal E} }^{\dagger }( {\mathcal E} {(\sigma )}^{-\tfrac{1}{2}}X {\mathcal E} {(\sigma )}^{-\tfrac{1}{2}}){\sigma }^{\tfrac{1}{2}}$. Here, ${\mathcal E}$ is the local von Neumann measurement ${{\rm{\Pi }}}_{A}\otimes {{\rm{\Pi }}}_{B}={({{\rm{\Pi }}}_{A}\otimes {{\rm{\Pi }}}_{B})}^{\dagger }$. Applied to the Eq. (12), the recovery condition says that

13 $$\begin{array}{rcl} {\mathcal R} ({{\rm{\Pi }}}_{A}\otimes {{\rm{\Pi }}}_{B}({\rho }_{AB})) & = & \sum _{i,j}\,\frac{{\sum }_{\alpha ,\beta }{\lambda }_{\alpha \beta }{|\langle i|{\psi }_{\alpha }\rangle |}^{2}{|\langle j|{\varphi }_{\beta }\rangle |}^{2}}{({\sum }_{\alpha ,\beta }{\lambda }_{\alpha \beta }{|\langle i|{\psi }_{\alpha }\rangle |}^{2})\,({\sum }_{\alpha ,\beta }{\lambda }_{\alpha \beta }{|\langle j|{\varphi }_{\beta }\rangle |}^{2})}\\  &  & \times ({\rho }_{A}^{\frac{1}{2}}|i\rangle \langle i|{\rho }_{A}^{\frac{1}{2}})\otimes ({\rho }_{B}^{\frac{1}{2}}|j\rangle \langle j|{\rho }_{B}^{\frac{1}{2}}).\end{array}$$

By letting Eqs (11) and (13) be equal, and pre- and post-multiplying by ${\rho }_{A}^{-\tfrac{1}{2}}\otimes {\rho }_{B}^{-\tfrac{1}{2}}$, we obtain

14 $$\begin{array}{c}\sum _{i,j}\,\tfrac{{\sum }_{\alpha ,\beta }{\lambda }_{\alpha \beta }{|\langle i|{\psi }_{\alpha }\rangle |}^{2}{|\langle j|{\varphi }_{\beta }\rangle |}^{2}}{({\sum }_{\alpha ,\beta }{\lambda }_{\alpha \beta }{|\langle i|{\psi }_{\alpha }\rangle |}^{2})\,({\sum }_{\alpha ,\beta }{\lambda }_{\alpha \beta }{|\langle j|{\varphi }_{\beta }\rangle |}^{2})}\,|i\rangle \langle i|\otimes |j\rangle \langle j|\\ \quad =\sum _{\alpha ,\beta }\,\tfrac{{\lambda }_{\alpha \beta }}{({\sum }_{\xi }{\lambda }_{\alpha \xi })\,({\sum }_{\gamma }{\lambda }_{\gamma \beta })}\,|{\psi }_{\alpha }\rangle \langle {\psi }_{\alpha }|\otimes |{\varphi }_{\beta }\rangle \langle {\varphi }_{\beta }|\mathrm{.}\end{array}$$

Remarkably, ${\rho }_{A}^{-\tfrac{1}{2}}$ and ${\rho }_{B}^{-\tfrac{1}{2}}$ are defined as

$${\rho }_{A}^{-\frac{1}{2}}\equiv \sum _{\alpha :{\sum }_{\beta }\,{\lambda }_{\alpha \beta }\ne 0}\,\frac{1}{\sqrt{\sum _{\beta }\,{\lambda }_{\alpha \beta }}}|{\psi }_{\alpha }\rangle \langle {\psi }_{\alpha }|,\quad {\rho }_{B}^{-\frac{1}{2}}\equiv \sum _{\beta :{\sum }_{\alpha }\,{\lambda }_{\alpha \beta }\ne 0}\,\frac{1}{\sqrt{\sum _{\alpha }{\lambda }_{\alpha \beta }}}|{\varphi }_{\beta }\rangle \langle {\varphi }_{\beta }|,$$

where all ${\sum }_{\beta }{\lambda }_{\alpha \beta }$ and |*ψ*_*α*_〉 are the eigenvalues and eigenvectors of *ρ*_*A*_, and all ${\sum }_{\alpha }{\lambda }_{\alpha \beta }$ and |*ϕ*_*β*_〉 are the same requirements of *ρ*_*B*_. Thus in Eq. (14) we exclude terms on either side which are not in the support of *ρ*_*A*_ and *ρ*_*B*_.

Let *i*′, *j*′, *α*′ and *β*′ be the values of *i*, *j*, *α* and *β* in Eqs (11) and (13). After taking the inner product $\langle i^{\prime} |\langle j^{\prime} |()|{\psi }_{\alpha ^{\prime} }\rangle |{\varphi }_{\beta ^{\prime} }\rangle$ on either side of Eq. (13), we have

15 $$\tfrac{{\sum }_{\alpha ,\beta }{\lambda }_{\alpha \beta }{|\langle i^{\prime} |{\psi }_{\alpha }\rangle |}^{2}{|\langle j^{\prime} |{\varphi }_{\beta }\rangle |}^{2}}{({\sum }_{\alpha ,\beta }{\lambda }_{\alpha \beta }{|\langle i^{\prime} |{\psi }_{\alpha }\rangle |}^{2})\,({\sum }_{\alpha ,\beta }{\lambda }_{\alpha \beta }{|\langle j^{\prime} |{\varphi }_{\beta }\rangle |}^{2})}\,\langle i^{\prime} |{\psi }_{\alpha ^{\prime} }\rangle \langle j^{\prime} |{\varphi }_{\beta ^{\prime} }\rangle =\tfrac{{\lambda }_{\alpha ^{\prime} \beta ^{\prime} }}{({\sum }_{\beta }{\lambda }_{\alpha ^{\prime} \beta })\,({\sum }_{\alpha }{\lambda }_{\alpha \beta ^{\prime} })}\,\langle i^{\prime\prime} |{\psi }_{\alpha ^{\prime} }\rangle \langle j^{\prime} |{\varphi }_{\beta ^{\prime} }\rangle \mathrm{.}$$

If $\langle i^{\prime} |{\psi }_{\alpha ^{\prime} }\rangle \langle j^{\prime} |{\varphi }_{\beta ^{\prime} }\rangle \ne 0$, Eq. (15) means that

16 $$\frac{{\sum }_{\alpha ,\beta }{\lambda }_{\alpha \beta }{|\langle i^{\prime} |{\psi }_{\alpha }\rangle |}^{2}{|\langle j^{\prime} |{\varphi }_{\beta }\rangle |}^{2}}{({\sum }_{\alpha ,\beta }{\lambda }_{\alpha \beta }{|\langle i^{\prime} |{\psi }_{\alpha }\rangle |}^{2})\,({\sum }_{\alpha ,\beta }{\lambda }_{\alpha \beta }{|\langle j^{\prime} |{\varphi }_{\beta }\rangle |}^{2})}=\frac{{\lambda }_{\alpha ^{\prime} \beta ^{\prime} }}{({\sum }_{\beta }{\lambda }_{\alpha ^{\prime} \beta })\,({\sum }_{\alpha }{\lambda }_{\alpha \beta ^{\prime} })}\mathrm{.}$$

Due to Eq. (16), we confirm that the left sides of (16) are the same for all *i*′ and *j*′ satisfying $\langle i^{\prime} |{\psi }_{\alpha ^{\prime} }\rangle \langle j^{\prime} |{\varphi }_{\beta ^{\prime} }\rangle \ne 0$ when we fix *α*′ and *β*′. As the same reason, the right sides of (16) are the same for all *α*′ and *β*′ satisfying $\langle i^{\prime} |{\psi }_{\alpha ^{\prime} }\rangle \langle j^{\prime} |{\varphi }_{\beta ^{\prime} }\rangle \ne 0$ when we fix *i*′ and *j*′.

Expanding Eq. (13) continuously, we obtain that

17 $$\begin{array}{rcl} {\mathcal R} ({{\rm{\Pi }}}_{A}\otimes {{\rm{\Pi }}}_{B}({\rho }_{AB})) & = & \sum _{i,j}\,\frac{{\sum }_{\alpha ,\beta }{\lambda }_{\alpha \beta }{|\langle i|{\psi }_{\alpha }\rangle |}^{2}{|\langle j|{\varphi }_{\beta }\rangle |}^{2}}{({\sum }_{\alpha ,\beta }{\lambda }_{\alpha \beta }{|\langle i|{\psi }_{\alpha }\rangle |}^{2})\,({\sum }_{\alpha ,\beta }{\lambda }_{\alpha \beta }{|\langle j|{\varphi }_{\beta }\rangle |}^{2})}\,\\  &  & \times (\sum _{\alpha }\,\sqrt{\sum _{\beta }\,{\lambda }_{\alpha \beta }}\langle {\psi }_{\alpha }|i\rangle |{\psi }_{\alpha }\rangle )\\  &  & \times (\sum _{\alpha }\,\sqrt{\sum _{\beta }\,{\lambda }_{\alpha \beta }}\,\langle i|{\psi }_{\alpha }\rangle \langle {\psi }_{\alpha }|)\otimes (\sum _{\beta }\,\sqrt{\sum _{\alpha }\,{\lambda }_{\alpha \beta }}\langle {\varphi }_{\beta }|j\rangle |{\varphi }_{\beta }\rangle )\\  &  & \times (\sum _{\beta }\,\sqrt{\sum _{\alpha }\,{\lambda }_{\alpha \beta }}\langle j|{\varphi }_{\beta }\rangle \langle {\varphi }_{\beta }|).\end{array}$$

By letting Eqs (11) and (17) be equal, we firstly consider the case that $|{\psi }_{{\alpha }_{1}}\rangle$ $(|{\varphi }_{{\beta }_{1}}\rangle )$ and all the other |*ψ*_*α*_〉 (|*ϕ*_*β*_〉) have disjoint coherence support. Without loss of generality, let {|*i*_1_〉, |*i*_2_〉} and {|*j*_1_〉, |*j*_2_〉} be the coherence support of $|{\psi }_{{\alpha }_{1}}\rangle$ and $|{\varphi }_{{\beta }_{1}}\rangle$, respectively. A litle thought shows that the sum of only the four terms (*i*_1_, *j*_1_), (*i*_1_, *j*_2_), (*i*_2_, *j*_1_) and (*i*_2_, *j*_2_) in Eq. (17) coincides with the term ${\lambda }_{{\alpha }_{1}{\beta }_{1}}|{\psi }_{{\alpha }_{1}}\rangle \langle {\psi }_{{\alpha }_{1}}|\otimes |{\psi }_{{\alpha }_{1}}\rangle \langle {\psi }_{{\alpha }_{1}}|$ in Eq. (11).

18 $$\begin{array}{l}\sum _{\begin{array}{c}i={i}_{1},{i}_{2};\\ j={j}_{1},{j}_{2}\end{array}}\,\tfrac{({\sum }_{\begin{array}{c}\alpha ={\alpha }_{1},{\alpha }_{2};\\ \beta ={\beta }_{1},{\beta }_{2}\end{array}}{\lambda }_{\alpha \beta }{|\langle i|{\psi }_{\alpha }\rangle |}^{2}{|\langle j|{\varphi }_{\beta }\rangle |}^{2})\,({\sum }_{\beta }{\lambda }_{{\alpha }_{1}\beta }+{\sum }_{\beta }{\lambda }_{{\alpha }_{2}\beta })\,({\sum }_{\alpha }{\lambda }_{\alpha {\beta }_{1}}+{\sum }_{\alpha }{\lambda }_{\alpha {\beta }_{2}})}{({\sum }_{\alpha ={\alpha }_{1},{\alpha }_{2};\beta }{\lambda }_{\alpha \beta }{|\langle i|{\psi }_{\alpha }\rangle |}^{2})\,({\sum }_{\alpha ;\beta ={\beta }_{1},{\beta }_{2}}{\lambda }_{\alpha \beta }{|\langle j|{\varphi }_{\beta }\rangle |}^{2})}\\ \begin{array}{c}\quad \times (\tfrac{{\sum }_{\beta }{\lambda }_{{\alpha }_{1}\beta }}{{\sum }_{\beta }{\lambda }_{{\alpha }_{1}\beta }+{\sum }_{\beta }{\lambda }_{{\alpha }_{2}\beta }}\,{|\langle i|{\psi }_{{\alpha }_{1}}\rangle |}^{2}|{\psi }_{{\alpha }_{1}}\rangle \langle {\psi }_{{\alpha }_{1}}|\\ \quad +\tfrac{{\sum }_{\beta }{\lambda }_{{\alpha }_{2}\beta }}{{\sum }_{\beta }{\lambda }_{{\alpha }_{1}\beta }+{\sum }_{\beta }{\lambda }_{{\alpha }_{2}\beta }}\,{|\langle i|{\psi }_{{\alpha }_{2}}\rangle |}^{2}|{\psi }_{{\alpha }_{2}}\rangle \langle {\psi }_{{\alpha }_{2}}|\end{array}\\ \begin{array}{c}\quad +\tfrac{\sqrt{({\sum }_{\beta }{\lambda }_{{\alpha }_{1}\beta })\,({\sum }_{\beta }{\lambda }_{{\alpha }_{2}\beta })}}{{\sum }_{\beta }{\lambda }_{{\alpha }_{1}\beta }+{\sum }_{\beta }{\lambda }_{{\alpha }_{2}\beta }}\,\langle {\psi }_{{\alpha }_{1}}|i\rangle \langle i|{\psi }_{{\alpha }_{2}}\rangle |{\psi }_{{\alpha }_{1}}\rangle \langle {\psi }_{{\alpha }_{2}}|\\ \quad +\tfrac{\sqrt{({\sum }_{\beta }{\lambda }_{{\alpha }_{1}\beta })\,({\sum }_{\beta }{\lambda }_{{\alpha }_{2}\beta })}}{{\sum }_{\beta }{\lambda }_{{\alpha }_{1}\beta }+{\sum }_{\beta }{\lambda }_{{\alpha }_{2}\beta }}\,\langle {\psi }_{{\alpha }_{2}}|i\rangle \langle i|{\psi }_{{\alpha }_{1}}\rangle |{\psi }_{{\alpha }_{2}}\rangle \langle {\psi }_{{\alpha }_{1}}|)\end{array}\\ \begin{array}{c}\quad \otimes (\tfrac{{\sum }_{\alpha }{\lambda }_{\alpha {\beta }_{1}}}{{\sum }_{\alpha }{\lambda }_{\alpha {\beta }_{1}}+{\sum }_{\alpha }{\lambda }_{\alpha {\beta }_{2}}}\,{|\langle j|{\varphi }_{{\beta }_{1}}\rangle |}^{2}|{\varphi }_{{\beta }_{1}}\rangle \langle {\varphi }_{{\beta }_{1}}|\\ \quad +\tfrac{{\sum }_{\alpha }{\lambda }_{\alpha {\beta }_{2}}}{{\sum }_{\alpha }{\lambda }_{\alpha {\beta }_{1}}+{\sum }_{\alpha }{\lambda }_{\alpha {\beta }_{2}}}\,{|\langle j|{\varphi }_{{\beta }_{2}}\rangle |}^{2}|{\varphi }_{{\beta }_{2}}\rangle \langle {\varphi }_{{\beta }_{2}}|\end{array}\\ \begin{array}{c}\quad +\tfrac{\sqrt{({\sum }_{\alpha }{\lambda }_{\alpha {\beta }_{1}})\,({\sum }_{\alpha }{\lambda }_{\alpha {\beta }_{2}})}}{{\sum }_{\alpha }{\lambda }_{\alpha {\beta }_{1}}+{\sum }_{\alpha }{\lambda }_{\alpha {\beta }_{2}}}\,\langle {\varphi }_{{\beta }_{1}}|j\rangle \langle j|{\varphi }_{{\beta }_{2}}\rangle |{\varphi }_{{\beta }_{1}}\rangle \langle {\varphi }_{{\beta }_{2}}|\\ \quad +\tfrac{\sqrt{({\sum }_{\alpha }{\lambda }_{\alpha {\beta }_{1}})\,({\sum }_{\alpha }{\lambda }_{\alpha {\beta }_{2}})}}{{\sum }_{\alpha }{\lambda }_{\alpha {\beta }_{1}}+{\sum }_{\alpha }{\lambda }_{\alpha {\beta }_{2}}}\,\langle {\varphi }_{{\beta }_{2}}|j\rangle \langle j|{\varphi }_{{\beta }_{1}}\rangle |{\varphi }_{{\beta }_{2}}\rangle \langle {\varphi }_{{\beta }_{1}}|).\end{array}\end{array}$$

Secondly, we consider the case that the coherence support of $|{\psi }_{{\alpha }_{1}}\rangle$ has some intersection with that of other |*ψ*_*α*_〉, or the coherence support of $|{\varphi }_{{\beta }_{1}}\rangle$ has some intersection with that of other |*ϕ*_*β*_〉. Without loss of generality, let {|*i*_1_〉, |*i*_2_〉} be the coherence support of $|{\psi }_{{\alpha }_{1}}\rangle$ and $|{\psi }_{{\alpha }_{2}}\rangle$, and the set of {|*i*_1_〉, |*i*_2_〉} has no intersection with the coherence support of other |*ψ*_*α*_〉 except $|{\psi }_{{\alpha }_{1}}\rangle$ and $|{\psi }_{{\alpha }_{2}}\rangle$. Similarly, let {|*j*_1_〉, |*j*_2_〉} be the coherence support of $|{\varphi }_{{\beta }_{1}}\rangle$ and $|{\varphi }_{{\beta }_{2}}\rangle$, and the set of {|*j*_1_〉, |*j*_2_〉} has no intersection with the coherence support of other |*ϕ*_*β*_〉 except $|{\varphi }_{{\beta }_{1}}\rangle$ and $|{\varphi }_{{\beta }_{2}}\rangle$. The sum of only the four terms (*i*_1_, *j*_1_), (*i*_1_, *j*_2_), (*i*_2_, *j*_1_) and (*i*_2_, *j*_2_) in Eq. (17) will be written as the above formula (18).

Using Eq. (16), we know that the formulas

$$\frac{{\sum }_{\begin{array}{c}\alpha ={\alpha }_{1},{\alpha }_{2};\\ \beta ={\beta }_{1},{\beta }_{2}\end{array}}{\lambda }_{\alpha \beta }{|\langle i|{\psi }_{\alpha }\rangle |}^{2}{|\langle j|{\varphi }_{\beta }\rangle |}^{2}}{({\sum }_{\alpha ={\alpha }_{1},{\alpha }_{2};\beta }{\lambda }_{\alpha \beta }{|\langle i|{\psi }_{\alpha }\rangle |}^{2})\,({\sum }_{\alpha ;\beta ={\beta }_{1},{\beta }_{2}}{\lambda }_{\alpha \beta }{|\langle j|{\varphi }_{\beta }\rangle |}^{2})},$$

are the same for any *i* = *i*_1_, *i*_2_ and *j* = *j*_1_, *j*_2_. Then, using the orthonormality of states in sets $\{|{\psi }_{{\alpha }_{1}}\rangle ,|{\psi }_{{\alpha }_{2}}\rangle \}$ and $\{|{\varphi }_{{\beta }_{1}}\rangle ,|{\varphi }_{{\beta }_{2}}\rangle \}$, and removing the cross terms that contain $|{\psi }_{{\alpha }_{1}}\rangle \langle {\psi }_{{\alpha }_{2}}|$, $|{\psi }_{{\alpha }_{2}}\rangle \langle {\psi }_{{\alpha }_{1}}|$, $|{\varphi }_{{\beta }_{1}}\rangle \langle {\varphi }_{{\beta }_{2}}|$ or $|{\varphi }_{{\beta }_{2}}\rangle \langle {\varphi }_{{\beta }_{1}}|$ in formula (18), we obtain the simplified form of formula (18):

19 $${p}_{{\alpha }_{1}{\alpha }_{2}{\beta }_{1}{\beta }_{2}}({\mu }_{1}|{\psi }_{{\alpha }_{1}}\rangle \langle {\psi }_{{\alpha }_{1}}|+\,{\mu }_{2}|{\psi }_{{\alpha }_{2}}\rangle \langle {\psi }_{{\alpha }_{2}}|)\otimes ({\eta }_{1}|{\varphi }_{{\beta }_{1}}\rangle \langle {\varphi }_{{\beta }_{1}}|+\,{\eta }_{2}|{\varphi }_{{\beta }_{2}}\rangle \langle {\varphi }_{{\beta }_{2}}|),$$

where *μ*_1(2)_, *η*_1(2)_ and ${p}_{{\alpha }_{1}{\alpha }_{2}{\beta }_{1}{\beta }_{2}}$ are non-negative, and satisfy

$$\begin{array}{rcl}\quad \,\,{\mu }_{\mathrm{1(2)}} & = & \tfrac{{\sum }_{\beta }{\lambda }_{{\alpha }_{\mathrm{1(2)}}\beta }}{{\sum }_{\beta }{\lambda }_{{\alpha }_{1}\beta }+{\sum }_{\beta }{\lambda }_{{\alpha }_{2}\beta }},\\ \quad \,\,{\eta }_{\mathrm{1(2)}} & = & \tfrac{{\sum }_{\alpha }{\lambda }_{\alpha {\beta }_{\mathrm{1(2)}}}}{{\sum }_{\alpha }{\lambda }_{\alpha {\beta }_{1}}+{\sum }_{\alpha }{\lambda }_{\alpha {\beta }_{2}}},\\ \,{\mu }_{1}+{\mu }_{2} & = & 1,\\ \,\,{\eta }_{1}+{\eta }_{2} & = & 1,\\ {p}_{{\alpha }_{1}{\alpha }_{2}{\beta }_{1}{\beta }_{2}} & = & \sum _{\begin{array}{c}\alpha ={\alpha }_{1},{\alpha }_{2};\\ \beta ={\beta }_{1},{\beta }_{2}\end{array}}\,{\lambda }_{\alpha \beta }\mathrm{.}\end{array}$$

What’s more, formula (19) coincides with the sum of partial terms in Eq. (11):

$$\sum _{\begin{array}{c}\alpha ={\alpha }_{1},{\alpha }_{2};\\ \beta ={\beta }_{1},{\beta }_{2}\end{array}}\,{\lambda }_{\alpha \beta }|{\psi }_{\alpha }\rangle \langle {\psi }_{\alpha }|\otimes |{\varphi }_{\beta }\rangle \langle {\varphi }_{\beta }|.$$

Finally, other cases that there exist some intersection of coherence support of |*ψ*_*α*_〉 or |*ϕ*_*β*_〉 can be discussed similarly, and the results like formula (19) will be obtained. Hence, the equality of Eqs (11) and (17) means that *ρ*_*AB*_ has a decomposition as given in Eq. (5) in the main text.□
